# Mobile health technology in quality assessment of pediatric ileocolonoscopy: Results of the SIGENP national program

**DOI:** 10.1055/a-2592-2914

**Published:** 2025-06-17

**Authors:** Salvatore Oliva, Giusy Russo, Lucia Cococcioni, Francesca Destro, Marco Deganello Saccomani, Claudia Banzato, Barbara Parma, Giulia Franchino, Giovanni Di Nardo, Emanuele Nicastro, Paolo Orizio, Emanuele Dabizzi, Giorgio Raffaele Fava, Andrea Chiaro, Maristella Pellegrino, Fabiola Fornaroli, Antonio Pizzol, Caterina Strisciuglio, Caterina Pacenza, Sara Renzo, Cosimo Ruggiero, Francesco Morotti, Lorenzo Norsa

**Affiliations:** 1 Pediatric Gastroenterology and Liver Unit, Maternal and Child Health Department, Sapienza University of Rome, Rome, Italy; 2 37220 Department of Pediatrics, Vittore Buzzi Children's Hospital, Milano, Italy; 3 37220 Department of Pediatric Surgery, Vittore Buzzi Children's Hospital, Milano, Italy; 4 Department of Pediatrics, Woman’s and Child’s University Hospital of Verona, Verona, Italy; 5 Department of Pediatrics, Mariani Foundation Center for Fragile Child, ASST-Lariana, Sant'Anna Hospital, San Fermo della Battaglia, Italy; 6 9311 NESMOS Department, University of Rome La Sapienza, Rome, Italy; 7 9333 Pediatric Hepatology Gastrosenterology and Transplantation Unit, ASST Papa Giovanni XXIII, Bergamo, Italy; 8 Department of Pediatric Surgery, Spedali Civili Children's Hospital, Brescia, Italy; 9 26217 Gastroenterology and Interventional Endoscopy Unit, Surgical Department, AUSL di Bologna, Bologna, Italy; 10 9339 Department of Pediatric Surgery, Fondazione IRCCS Ca' Granda Ospedale Maggiore Policlinico, Milan, Italy; 11 18572 Pediatric Gastroenterology and Endoscopy Unit, IRCCS Istituto Giannina Gaslini, Genova, Italy; 12 9338 Pediatric Surgery Department, ASST Grande Ospedale Metropolitano Niguarda, Milano, Italy; 13 9370 Gastroenterology and Endoscopy Unit, Department of Medicine and Surgery, University of Parma, Parma, Italy; 14 472627 Pediatric Gastroenterology Unit, Regina Margherita Children's Hospital, Turin, Italy; 15 507855 Department of Woman, Child, General and Specialistic Surgery, University of Campania Luigi Vanvitelli School of Medicine and Surgery, Napoli, Italy; 16 Department of Pediatrics, San Giovanni di Dio Hospital, Crotone, Italy; 17 Gastroenterology and Nutrition Unit, Meyer Children’s Hospital, Florence, Italy; 18 60252 Division of Pediatrics, Department of Health Sciences, Università degli Studi del Piemonte Orientale Amedeo Avogadro Facoltà di Medicina e Chirurgia, Novara, Italy; 19 Neonatology and Neonatal Intensive Care Unit, Spedali Civili Children's Hospital, Brescia, Italy; 20 9333 Pediatric Hepatology Gastroenterology and Transplantation, ASST Papa Giovanni XXIII, Bergamo, Italy; 21 37220 Department of Pediatrics, Vittore Buzzi Children's Hospital, Vittore Buzzi Children's Hospital, Milano, Italy

**Keywords:** Quality and logistical aspects, Quality management, Endoscopy Lower GI Tract, Pediatric endoscopy

## Abstract

**Background and study aims:**

Currently, there is no formal quality assessment of pediatric gastrointestinal endoscopy. We innovatively used mobile health (mHealth) technology to determine the quality of pediatric ileocolonoscopy (IC) in Italy.

**Methods:**

Between April 2019 and March 2021, we prospectively collected data (demographics, procedure information, pre/intra/post-procedure indicators, patient satisfaction questionnaires) from the Italian Society of Pediatric Gastroenterology, Hepatology, and Nutrition using the ENDO-PED mobile app.

**Results:**

Of 3410 registered procedures, 827 ICs were analyzed. Mean patient age was 11.1 ± 4.7 years. The most frequent indication was IBD follow-up or diagnosis (57.9%). Therapeutic ICs accounted for 11%, with polypectomy being the most common procedure. Among pre-procedure indicators, waiting time < 30 days was reported in 70.7%, informed consent was signed in 99.8% of cases, and 90.8% of patients completed > 90% of bowel preparation. In terms of intra-procedure indicators, deep sedation was the most commonly used method (77.8%). A high level of bowel cleansing was achieved in 87.4% of patients, with a terminal ileal (TI) intubation rate of 91.6%. Mean IC time with and without TI intubation was 24.2 ± 15.5 and 22.6 ± 15.6 minutes, respectively ( *P* =0.2). Regarding post-procedure indicators, late complications occurred in three children (0.4%), and a final report was issued in 96% of cases, with 67.2% being completed after more than 15 days.

**Conclusions:**

mHealth was effective in assessing the quality of pediatric endoscopy. Levels of bowel preparation, sedation, TI intubation rate, and safety were adequate in Italy, whereas waiting time and post-procedure communication seemed to be the most critical areas of concern.

## Introduction

The importance of measuring healthcare quality is widely acknowledged, because outcome data can significantly enhance care delivery 1 2 . With respect to gastrointestinal endoscopy, the inception of colorectal cancer screening programs has prompted development of quality and safety indicators 3 4 5 . However 6 7 8 , evidence in pediatric gastrointestinal endoscopy remains limited 9 10 . High-quality pediatric endoscopy is achieved when patients undergo indicated procedures safely and efficiently in appropriate settings, ensuring accurate diagnosis or exclusion, and receiving appropriate therapy while minimizing risks 11 . Nevertheless, adult guidelines tailored for adult patients cannot be directly applied to pediatric endoscopy.

In 2017, experts from the North American Society of Pediatric Gastroenterology (NASPGHAN) published an opinion-based framework 12 to improve quality within pediatric endoscopy units as a first attempt to fill the gap. However, the evidence of a substantial lack of literature on quality in pediatric endoscopy made the gathering of a broader expert consensus imperative. This ultimately led to establishment of the Pediatric Endoscopy Quality Improvement Network (PEnQuIN) in 2018.

Comprising experts from North American and European pediatric gastroenterology societies (NASPGHAN, European Society for Paediatric Gastroenterology, Hepatology, and Nutrition), PEnQuIN was dedicated to defining minimum expected pediatric endoscopy quality standards and identifying key quality indicators 13 . According to current guidelines, therefore, an appropriate indication for colonoscopy, adequate waiting times, a complete examination including inspection of the cecum and especially the terminal ileum, adequate bowel preparation, absence of complications, and proper communication with the patient and parents/caregivers during the pre-procedural and post-procedural phases are among the main quality indicators in pediatric ileocolonoscopy. After publication of the set of PEnQuIN guidelines, assessing the quality of pediatric endoscopy activity has become an open field for research, with several knowledge gaps to be bridged 11 14 15 16 .

Although awareness of the need for quality assessment in pediatric endoscopy is growing, a significant gap in literature on the subject persists. One of the limiting factors was the difficulty in collecting real-world data from pediatric endoscopy units. Adoption of electronic data collection represented a significant advancement in terms of time efficiency and data quality 17 . However, despite these benefits, the conventional desktop-based, second-stage electronic collection method proved cumbersome in the pediatric endoscopy setting. In recent years, smartphones have emerged as a more practical tool for longitudinal data collection and quantitative research 18 19 20 .

In collaboration with the Endoscopy Special Interest Group of the Italian Society of Pediatric Gastroenterology (SIGENP), we developed a smartphone-based application for streamlined data collection, innovatively utilizing mobile health (m-health) technology. Consequently, we undertook the first-ever assessment of the activities of Italian pediatric endoscopy centers. Quality indicators were derived from the 2017 report of Kramer and colleagues 12 and later merged into the definitive PEnQuIN quality guidelines, which were in the process of being chosen at the commencement of the project. Results of the quality indicators for upper endoscopy have already been published 21 . Here, we present findings on lower endoscopic procedures.

## Patients and methods

### Objectives

This study aimed primarily to evaluate adherence of pediatric endoscopy centers to organizational and procedural quality standards, with secondary objectives focused on identifying areas for quality improvement.

### Study design and setting

Conducted as a prospective observational survey, this study included all patients undergoing endoscopic procedures at Italian endoscopy centers, classified as facilities specializing in pediatric endoscopy or adult gastroenterology centers conducting endoscopies on children. Data analysis concentrated exclusively on lower endoscopy, specifically ileocolonoscopy (IC), rectosigmoidoscopy (RCS), and retrograde enteroscopy. Procedures beyond visual inspection or biopsy, termed "operative" procedures, were specifically scrutinized.

### Data collection

We collected prospective data using a mobile application named EndoPed, designed by principal investigators (PIs: SO and LN) and developed by the Endoscopy Special Interest Group of SIGENP with the assistance of a software designer. Operating within the Google Base environment, EndoPed employed a NoSQL database structure and adhered to the General Data Protection Regulation (GDPR) European laws. All SIGENP pediatric endoscopy centers were invited to participate, including all procedures performed between April 1, 2019, and March 30, 2021. Each operator was provided login credentials (email and password) and required to complete a profile upon first login. General information, pre-, intra-, and post-procedural quality indicators, and a patient satisfaction questionnaire were anonymously collected for each procedure by assigning a unique identification code.

### Operator profile

The following information was collected in each operator’s profile section: specialty profile (pediatrics, pediatric surgery, or adult gastroenterology), years of experience, place of work, and average number of endoscopies performed per year in the referral center and by the operator.

### Procedure information

The following information was collected for each procedure: identification number and date of the procedure, patient demographics (age and sex), indications for the procedure, type of procedure, and whether it was operative or nonoperative.

### Quality indicators

Pre-procedure indicators included events between the indication and the performance of endoscopy, such as informed consent acquisition, waiting time, time to explain the procedure, and type of bowel preparation.

Intra-procedure indicators were events within the time interval from insertion of the instrument (or administration of sedation) to its removal, including sedation type, duration, Boston Bowel Preparation Scale (BBPS) 22 , bowel biopsies, intra-procedural adverse events (AEs), and terminal ileum (TI) intubation.

Post-procedure indicators were events occurring within 14 days after the examination, including AEs, a modified version of the Group Health Association of America-9 (GHAA-9m) satisfaction questionnaire, and waiting time for microscopic examination.

Participating researchers designed, approved, and analyzed the study protocol. All identifiable medical information was removed, and all analyses were performed using anonymized data. Data collection adhered to good clinical practice policies and the GDPR 2016/679. Written informed consent was obtained from all subjects or their parents/legal representatives. The study protocol was defined in accordance with the Declaration of Helsinki and approved by the ethics committee of Policlinico Universitario Umberto I in Rome (ref 4771/21). All authors reviewed the study data and approved the final manuscript.

### Statistical analysis

Statistical data were processed using frequencies (N) and percentages (%) to summarize categorical variables, whereas quantitative variables were summarized using means and standard deviation (SD) or median and interquartile range (IQR), as appropriate. Categorical and continuous variables between groups or independent samples were compared using the Pearson’s chi square test and *t* test, respectively. *P* < 0.05 was considered to be statistically significant. Data are presented as “mean-SD” or “N-(%)”. All analyses were performed using SPSS v.22.0 (IBM Corp., Armonk, New York, United States).

## Results

A total of 3582 procedures were registered from participating centers ( Table 1 ). Among these, 875 (24%) were lower endoscopies: 827 (94.5%) ICs and 48 (5.5%) RCSs. The database underwent a thorough clinical review to identify outliers and address missing data. The mean age for these patients was 11.1 ± 4.7 years, with approximately half of the procedures (50.6%) performed on female patients. The median number of procedures performed per center was 31 (range: 5–67), with no statistically significant difference in distribution. Of the procedures, 665 (80.4%) were performed by a pediatric gastroenterologist, 105 (12.7%) by a pediatric surgeon, and 57 (6.9%) by an adult gastroenterologist. Among the 736 ICs (89%) that were nonoperative, chronic inflammatory bowel disease (IBD) was the main indication for diagnosis and follow-up in 41.3% and 16.6% of cases, respectively. Therapeutic IC was performed in 11%, with polypectomy being the most common procedure (74.7% of operative IC) ( Table 2 ). As for RCSs, 5 (10.4%) were performed in infants with hematochezia, in suspected allergic proctocolitis and 43 (89.6%) in patients with ulcerative colitis flare-ups.

**Table:** **Table 1** Distribution of endoscopic procedures by center.

Center	Procedures, n (%)
Bergamo	44 (5.32)
Bologna – Maggiore Hospital	31 (3.75)
Como	7 (0.85)
Crotone	4 (0.48)
Florence - Meyer Children’s Hospital	80 (9.67)
Florence – Sant’Anna	1 (0.12)
Genoa – Gaslini Hospital	53 (6.41)
Messina	1 (0.12)
Milan – Buzzi Hospital	84 (10.16)
Milan – Niguarda Hospital	20 (2.42)
Milan - Policlinico	46 (5.56)
Naples	12 (1.45)
Novara	1 (0.12)
Parma	5 (0.60)
Roma – Bambino Gesù Children Hospital	5 (0.60)
Rome – Sapienza University (Policlinico Umberto I)	203 (24.55)
Rome – Sapienza University (Sant’Andrea Hospital)	102 (12.33)
Turin	86 (10.40)
Verona	42 (5.08)

**Table:** **Table 2** Indications for ileocolonoscopies, categorized into diagnostic and operative.

Indication	Procedures, n (%)
Abdominal pain	55 (6.65)
Abdominal pain + chronic diarrhea	16 (1.93)
Anemia	12 (1.45)
Chronic diarrhea	19 (2.30)
IBD	329 (39.78)
Juvenile polyposis	26 (3.14)
Other	80 (6.97)
Peutz-Jeghers syndrome	10 (1.21)
Proctorrhagia	14 (1.69)
Rectorrhagia	104 (12.58)
Suspected IBD	132 (15.96)
UNK	30 (3.63)
Therapeutic procedures	91 (11.00)
Clips	1 (1.10)
Hemostasis	1 (1.10)
Dilatation	5 (5.49)
Other	16 (17.58)
Polypectomy	68 (74.73)
IBD, inflammatory bowel disease; UNK, unknown.	

### Pre-procedure indicators

Waiting time < 15 days was reported in 32.6% of procedures, 15 to 30 days in 38.1%, 30 to 45 days in 9.1%, 45 to 60 days in 12.2%, and > 60 days in 8.0%. In eight cases (0.9%), colonoscopy was rescheduled due to patient needs. Informed consent was signed by 99.8% of patients and provided orally by two patients (0.2%). Healthcare staff took approximately 5 minutes to explain the procedure in 34.5% of cases, 10 minutes in 48.9%, 15 minutes in 13.6%, and 20 minutes in 2.9%.

Adequate bowel preparation (intake > 90% of the expected bowel preparation) was achieved by 90.8% of patients, with 4.0%, 2.8%, 1.7%, and 0.7% of patients taking 76% to 90%, 51% to 75%, 25% to 50%, and < 25% of the expected preparation, respectively. Oral intake was sufficient for 97% of children, whereas 3% required a nasogastric tube for completion. Among these, 8.8% took polyethylene glycol (PEG) with electrolytes, 35.8% took PEG alone, 55.3% took sodium picosulfate/magnesium citrate (PICO), and 0.1% took senna syrup.

### Intra-procedural indicators

Deep sedation was the most used method (77.8%), followed by general anesthesia (16.3%) and conscious sedation (5.9%). The most frequently administered drugs were propofol, sevoflurane, midazolam, and fentanyl, either individually or in various combinations.

**Fig. 1:**
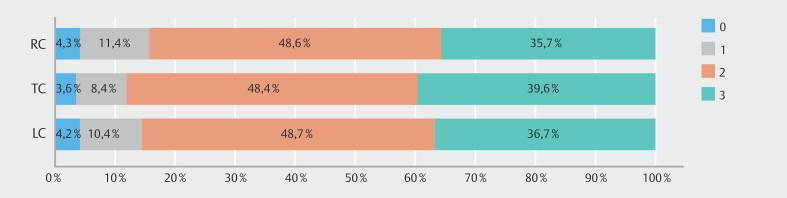
Boston Bowel Preparation Scale (BBPS) scores for each segment. BBPS, Boston Bowel Preparation Scale; RC, right colon; TC, transverse colon; LC, left colon.

Mean BBPS score value was 2.18 ± 0.78, 2.24 ± 0.75, and 2.16 ± 0.78 for the left, transverse, and right colon, respectively, with a BBPS score ≥ 5 reported in 723 children (87.4%) ( Fig. 1 ). Quality of bowel preparation was not correlated with age ( *P* = 0.8). Adequate bowel preparation ranged from 60% to 100%, with statistically significant differences among the 19 centers ( *P* < 0.001).

**Fig. 2:**
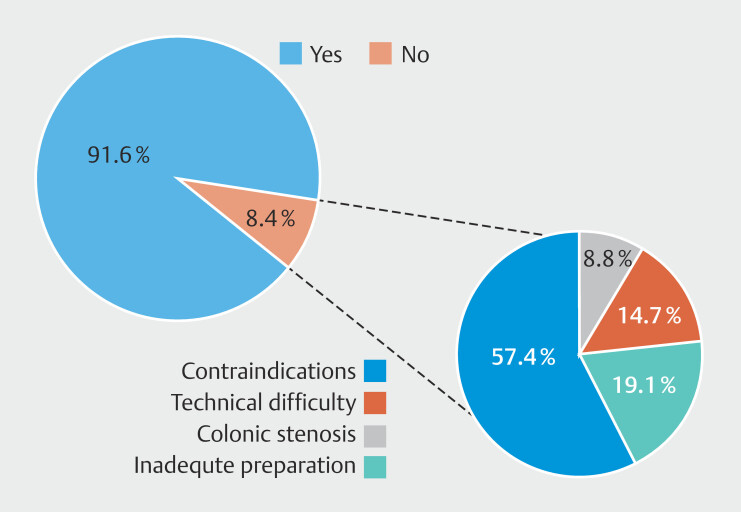
Rate of terminal ileum intubation.

The overall rate of TI intubation was 91.6%, with failure to achieve TI due to clinical contraindications in 57.4% of cases (anesthesia need or colonic mucosa too fragile), colonic stenosis in 8.8%, technical difficulty in 14.7%, and inadequate preparation in 19.1% ( Fig. 2 ). Patients aged < 5 years had a significantly lower ileal intubation rate compared with those aged 6 years or older (81.37% vs 92.4%; *P* < 0.001). Colonoscopies performed under conscious sedation had a significantly lower intubation rate (45.8%) compared with those under deep sedation and general anesthesia (93.9% and 91.1%, respectively; *P* < 0.0001). Colonoscopies with BBPS < 5 reported a lower TI intubation rate compared with those with BBPS ≥ 5 (76.5% vs 92.6%; *P* < 0.0001). TI intubation rates varied among centers and operators ( *P* < 0.0001), with no significant influence from operator experience or number of procedures performed annually.

**Fig. 3:**
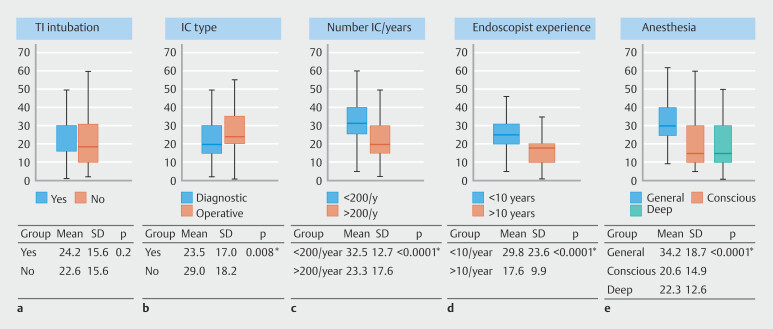
Mean procedure duration in minutes based on a ileal intubation, b type of colonoscopy, c number of colonoscopies performed per year per center, d experience of endoscopist, and e type of anesthesia used. TI, terminal ileum; IC, ileocolonoscopy.

Overall mean duration of IC was 24.1 ± 17.3 minutes, with no significant difference observed between IC with and without successful TI intubation (24.2 ± 15.5 vs 22.6 ± 15.6 minutes; *P* = 0.2). IC duration did not significantly vary with patient age (p = 0.8) or bowel preparation quality ( *P* = 0.2), but therapeutic IC procedures were longer than diagnostic ones (29.0 ± 18.2 vs 23.5 ± 17.0 minutes; *P* = 0.008). IC performed under general anesthesia had a longer duration (34.2 ± 18.7 minutes) compared with those under conscious sedation (20.6 ± 14.9 minutes; *P* < 0.001) or deep sedation (22.3 ± 12.6 minutes; *P* < 0.001). Mean IC duration varied significantly among centers ( *P* < 0.001), with shorter durations observed in centers performing at least 200 colonoscopies annually (23.3 vs 32.5 minutes; *P* < 0.0001) and when performed by endoscopists with ≥ 10 years of experience (17.6 vs 29.8 minutes; *P* < 0.0001) ( Fig. 3 ).

Intra-procedure complications occurred in five children (0.6%): four had anesthesiologic complications (3 cases of respiratory distress and one laryngospasm), and one experienced bowel perforation following colonic stenosis dilatation, which was successfully repaired with Endoclip placement.

Biopsies were performed in 71.3%, 62.5%, 45.8%, 67.2%, 43.4%, 73.8%, and 62.5% of cases in the ileum, cecum, ascending colon, transverse colon, descending colon, sigmoid colon, and rectum, respectively. Biopsy sampling met international guidelines 23 24 in 93.2%, 88%, 83.4%, 89.2%, 81.1%, 84.4%, and 88% of cases for each respective site.

### Post-procedure indicators

Late complications (occurring from 12 hours to 14 days) were observed in three children (0.4%), with one occurring after a therapeutic procedure: one had fever for 12 hours and two reported abdominal discomfort with need for medical reevaluation. A final report, including biopsy results, was provided to 794 families (96%), whereas the remaining 33 families (4%) did not attend the post-endoscopy follow-up. Communication with families regarding these results occurred within varying timeframes: less than 5 days (1.7%), 5 to 10 days (9.4%), 10 to 15 days (21.8%), and more than 15 days (67.2%). Of 475 patients (57.4%) who completed the satisfaction questionnaire, 6% did not respond positively.

## Discussion

This study represents the first comprehensive analysis of the quality of pediatric IC in Italy. With a satisfactory number of centers involved and a significant volume of procedures performed, the data collected provide a comprehensive overview of pediatric endoscopic activity in the country.

Analysis of IC distribution revealed wide variability in the number of procedures across individual centers, with the majority (67% of the total) conducted by only five centers, most of them located in northern or central Italy. These centers serve as referral centers for pediatric gastroenterology in highly populated urban areas. Notably, data collection occurred during the COVID-19 epidemic, which impacted pediatric endoscopy activity, especially in smaller centers and those most affected by the pandemic 25 26 . However, utilization of mobile health technology proved particularly effective in assessing the quality of pediatric endoscopy, facilitating real-time data collection in a simple, quick, and easily processable manner 27 28 , allowing for a comprehensive understanding of both positive and negative outcomes of IC in Italy.

Our study achieved an optimal overall rate of TI intubation, with statistically significant differences observed between centers and operators. However, these differences were not dependent on endoscopist experience or center volume. Recent studies 29 30 have shown that more recently trained endoscopists and those continuing in training programs achieve higher rates of ileal intubation 15 . These findings underscore the critical importance of enhancing the technical skills of pediatric endoscopists and emphasize the necessity for ongoing training and skill development programs tailored specifically to pediatric endoscopy. By investing in advanced training methodologies and providing opportunities for hands-on experience and quality indicator assessment, healthcare institutions can empower endoscopists to navigate complex procedures with greater precision and efficiency. However, PEnQuIN 11 13 has defined an ileal intubation rate of 85% or higher as a minimum target for high-quality endoscopy, a target met by most operators and centers in our study. TI intubation is of particular importance in pediatric gastrointestinal endoscopy 31 , because the main indication for procedures in children is IBD 13 15 . This also was confirmed in our study, where the main indication for IC was IBD, followed by bleeding and polyposis syndromes.

Our analysis revealed lower rates of ileal intubation in younger children, procedures performed under conscious sedation, and cases of inadequate bowel preparation. Poor bowel preparation, observed in a small percentage of patients, can significantly hinder the success of IC in children, affecting mucosal visibility and increasing risk of complications 6 32 33 . However, our data clearly show that the lower TI intubation rate in children younger than age 5 years is not related to quality of bowel preparation, because no difference in BBPS was found across age groups. This result is consistent with findings from other studies, which reported a lower rate of TI intubation in children younger than age 5 years, likely due to anatomical factors 32 33 .

The type of bowel preparation regimen varied in our sample, with the most common being PICO followed by PEG with or without electrolytes. Although there are no standard methods for pediatric bowel preparation 11 23 34 , recommendations from major pediatric gastroenterology societies advocate for low-volume preparations, such as PEG plus ascorbate or PICO plus magnesium citrate 35 .

Our data also indicated that lower BBPS scores were associated with longer procedure times, although statistical significance was not reached. However, median duration of procedures in our study was notably shorter than reported in other North American pediatric studies 29 32 36 . Moreover, differences in BBPS among the centers are likely due to several factors, such as varying preparation regimens and differing compliance with bowel preparation. These differences may stem from the distinct organizational structures of the centers, which perform IC in different settings (inpatient, day care, or outpatient), with varying levels of oversight by healthcare personnel.

Our centers complied with international guidelines 11 13 , recommending that no procedure be performed without proper sedation. The most commonly used sedative regimens combined a narcotic analgesic with a benzodiazepine, whereas propofol was the main sedative in deep sedation and general anesthesia regimens 37 38 39 . Regarding safety, both intra-procedural and post-procedural AEs were minimal, suggesting that pediatric colonoscopy in Italy is a safe procedure.

However, our study identified areas for improvement, notably in reducing waiting times for procedures and enhancing patient satisfaction. Actually, based on these results, SIGENP has already launched some national programs aimed at improving the quality of pediatric IC, especially with regard to variables that depend on healthcare personnel. Unfortunately, however, long pre-procedure and post-procedure waiting times often depend on the organization of the national healthcare system and improving them would require greater awareness from the political class.

As for patient satisfaction, it is necessary to first understand the patient perspective, which often differs from that of the physician. Indeed, as reported in the manuscript on upper endoscopic procedures, inclusion of a satisfaction questionnaire as a routine component of the endoscopy procedure would be desirable 21 . The study is subject to several limitations. First of all, the project commenced prior to final publication of the PEnQuIN quality standards. Consequently, the identified items only represent a subset of those later included in the final guidelines. Second, there was a suboptimal response and procedure submission rate from centers, which may have introduced a selection bias impacting generalizability of our findings. The survey aimed to reach all approximately 44 active pediatric endoscopy centers in Italy 40 . Although participant centers were strongly encouraged to contribute at least 50% of their annual workload to the research, the survey faced significant challenges due to the profound impact of the COVID-19 pandemic on the healthcare system, resulting in nearly halving endoscopy activity in Italy 25 . Despite these challenges, the survey achieved a 55% center participation rate with a median procedure submission rate of 30%. Although the impact of COVID-19 reduced the total number of procedures, particularly in regions most affected by the pandemic, it did not compromise generalizability of our results. Partial availability of data for some quality indicators further adds to limitations of the data analysis. Nonetheless, we believe our sample offers a reasonable snapshot of pediatric endoscopy in Italy.

## Conclusions

In conclusion, our study demonstrates the effectiveness of m-Health technology in evaluating quality of pediatric digestive endoscopy in Italy. While achieving satisfactory levels of bowel preparation, sedation, terminal ileum intubation rate, and safety, efforts must be directed toward reducing waiting times and enhancing patient satisfaction to further improve quality of care provided to pediatric patients and their families.
